# Efficacy of percutaneous versus intradermal BCG in the prevention of tuberculosis in South African infants: randomised trial

**DOI:** 10.1136/bmj.a2052

**Published:** 2008-11-13

**Authors:** Anthony Hawkridge, Mark Hatherill, Francesca Little, Margaret Ann Goetz, Lew Barker, Hassan Mahomed, Jerald Sadoff, Willem Hanekom, Larry Geiter, Greg Hussey

**Affiliations:** 1 South African Tuberculosis Vaccine Initiative, University of Cape Town, South Africa; 2 Department of Statistical Science, University of Cape Town; 3 Aeras Global TB Vaccine Foundation, Rockville, MD, USA

## Abstract

**Objective** To compare the incidence of tuberculosis over two years in infants vaccinated at birth with intradermal BCG or with percutaneous BCG.

**Design** Randomised trial.

**Setting** South Africa.

**Participants** 11 680 newborn infants.

**Interventions** Infants were randomised by week of birth to receive Tokyo 172 BCG vaccine through the percutaneous route (n=5775) or intradermal route (n=5905) within 24 hours of birth and followed up for two years.

**Main outcome measures** The primary outcome measure was documented *Mycobacterium tuberculosis* infection or radiological and clinical evidence of tuberculosis disease. Secondary outcome measures were rates of adverse events, all cause and tuberculosis specific admissions to hospital, and mortality.

**Results** The difference in the cumulative incidence of definite, probable, and possible tuberculosis between the intradermal group and the percutaneous group, as defined using study definitions based on microbiological, radiological, and clinical findings was −0.36% (95.5% confidence interval −1.27% to 0.54%). No significant differences were found between the routes in the cumulative incidence of tuberculosis using a range of equivalence of “within 25%.” Additionally, no significant differences were found between the routes in the cumulative incidence of adverse events (risk ratio 0.98, 95% confidence interval 0.91 to 1.06), including deaths (1.19, 0.89 to 1.58).

**Conclusion** Equivalence was found between intradermal BCG vaccine and percutaneous BCG in the incidence of tuberculosis in South African infants vaccinated at birth and followed up for two years. The World Health Organization should consider revising its policy of preferential intradermal vaccination to allow national immunisation programmes to choose percutaneous vaccination if that is more practical.

**Trial registration **ClinicalTrials.gov NCT00242047.

## Introduction

According to the World Health Organization 8.9 million new cases of tuberculosis and 1.7 million deaths from the disease occurred in 2004. Tuberculosis kills or debilitates more people aged between 15 and 59 than any other disease and accounts for 26% of all preventable deaths. More women of childbearing age in developing countries die from tuberculosis than from any other pregnancy or childbirth associated causes. The global annual incidence of tuberculosis continues to increase in Africa because of the HIV epidemic, whereas in all other regions it is stable or falling. The effectiveness of the only licensed vaccine for tuberculosis, Bacille Calmette-Guerin (BCG), is only partial.^1^ ^2^

BCG vaccine is given to nearly all newborn infants or young children in most developing countries, because it is thought to provide 80% protection against tuberculous meningitis and miliary disease, but protection against lung tuberculosis is highly variable.^3^ ^4^ Since its first use in humans in 1921, BCG has been given by a variety of routes, including by mouth, by intradermal injection, and percutaneously using a multipuncture injection device.

Currently the intradermal route is most commonly used. It is the only method recommended by WHO.^5^ Compared with the percutaneous route the intradermal route is said to deliver a more precise dose of vaccine,^6^ is cheaper,^7^ and may also induce higher rates of tuberculin skin test conversion. Skin test conversion is, however, a poor correlate of BCG induced protection against tuberculosis. Variable application techniques and differing devices may alter the number of bacilli given and possibly change the response to vaccination.^5^ ^8^ ^9^ ^10^ ^11^ Early studies have claimed that vaccination by the intradermal route induces a “better” immune response than by the percutaneous route. These findings are questioned by our recent reports on higher frequencies of BCG specific interferon-γ producing T cells; greater secretion of interferon-γ, tumour necrosis factor α, and interleukin 2; lower secretion of interleukin 4; and greater CD4 and CD8 T cell proliferation after percutaneous vaccination compared with intradermal vaccination.^12^ ^13^

Some countries prefer percutaneous BCG because of concerns about adverse events associated with the intradermal route, including pain, inflammatory changes,^14^ and abscesses.^15^ One such country was Japan, which switched to using percutaneous BCG in 1966 as those responsible thought this route was less reactogenic than the intradermal route.^16^ As of 2000, Japan and South Africa were the only two countries with large BCG vaccination programmes that still used the percutaneous route.

In 1999, following a consultative meeting, the South African National Department of Health decided to change from the percutaneous route to intradermal route to fall in line with WHO recommendations. Consensus was, however, lacking as to which method was better for protection against clinical tuberculosis and complications of the disease. Therefore, along with a recommendation to change vaccination policy the meeting recommended that additional research on methods of BCG vaccination should be carried out. We carried out a randomised trial to compare the incidence of tuberculosis over two years in infants vaccinated at birth with intradermal BCG or with percutaneous BCG.

## Methods

We carried out the study in a rural region of South Africa, located 100 km north east of Cape Town. In 2001 the study area had a population of 310 860, a reported annual incidence of tuberculosis in adults of 515 per 100 000, a reported HIV seroprevalence in pregnant women attending public antenatal clinics ranging from 5.7% to 6.2%, and an estimated rate of tuberculosis in under 5s of 2.5% per annum.^17^ ^18^

We included infants if they were born at one of the five public obstetric units in the study area, were eligible to receive routine BCG vaccination within the first 24 hours of birth, and their mothers were resident in the study area.

### Interventions

The BCG Laboratory in Tokyo, Japan, provided preparations of the Tokyo 172 BCG substrain vaccine. We chose this vaccine because it is the only commercially available product that comes in both percutaneous and intradermal formulations. The vaccine was given according to manufacturer’s instructions and the dosage determined and justified by the manufacturer. Intradermal BCG was given in a dose of 0.05 ml (20×10^6^ colony forming units/ml) from a single dose plastic syringe through a short bevelled 10 mm 25 gauge intradermal needle, into the lower deltoid region of the right arm at the insertion point of the muscle. For the percutaneous vaccine we evenly spread one drop of solution, containing 80 mg/ml BCG (3×10^9^ colony forming units/ml), over the skin and, using a nine pronged vaccination tool (BCG Laboratory), perforated the skin three quarters of the way up the right upper arm in the deltoid area, and a second time about 2 cm more distally. The percutaneous product needs to be given at a titre about 1.5 times higher than the intradermal product to achieve similar results (tuberculin skin test conversion). The infants were vaccinated within 24 hours of birth.

### Outcome measures

Our primary outcome was the incidence of tuberculous events in infants after vaccination with Tokyo 172 BCG administered by either the percutaneous route or the intradermal route. We considered events with bacteriological or histological confirmation or that met strict clinical criteria, occurring in the two years after birth. Our null hypothesis was that there would be a difference in the incidence of tuberculosis between the groups on the basis of a prespecified margin for equivalence.

Our secondary outcomes were the rates of adverse events, including local and systemic ones related to vaccination, admissions to hospital for all causes and for tuberculosis, and mortality. A further objective of this trial was to show the capacity to do a vaccine trial in this environment according to the standards expected of the International Conference on Harmonisation and WHO good clinical practice.

### Quality of measurements

If we suspected tuberculosis in any of the infants before age 2 years on the basis of a history of contact or because they exhibited signs or symptoms of the disease, we admitted them to a ward dedicated to verification of the disease. A study doctor and, when appropriate, study nurses carried out a clinical examination, chest radiography, tuberculin skin testing, HIV testing, and both gastric aspiration and sputum induction for mycobacterial culture. Confirmed tuberculosis was categorised as definite, probable, or possible using a diagnostic algorithm (fig 1). Infants who tested positive for HIV were referred for further evaluation and management to a specialist hospital.

**Figure fig1:**
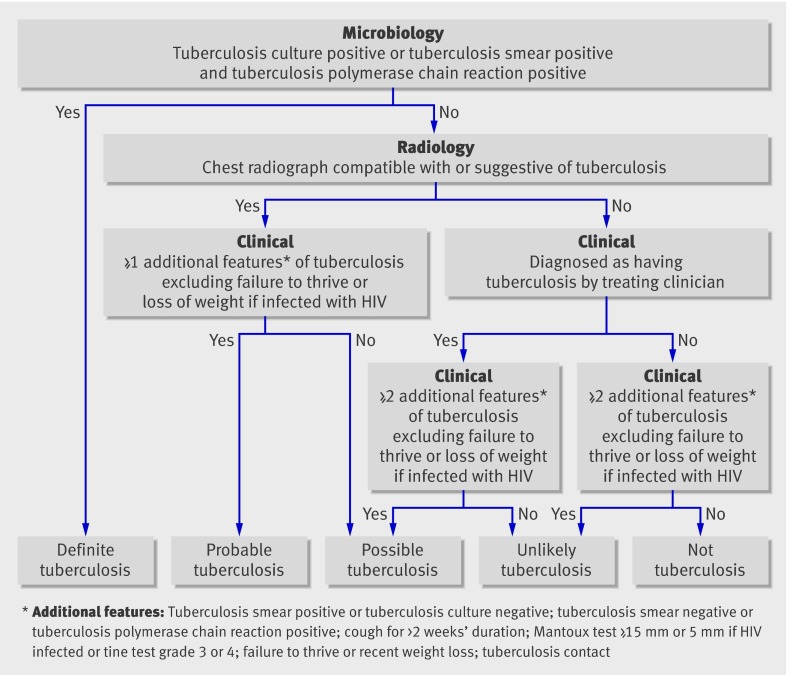
**Fig 1** Diagnostic algorithm for confirmation of tuberculosis in infants. Those who underwent investigations on the ward were admitted directly from their homes and were relatively well. Those requiring in-patient treatment for unrelated conditions were admitted for investigation of tuberculosis after hospital discharge and resolution of coexistent illness

The algorithm classified suspected cases into definite, probable, possible, and unlikely. Definite tuberculosis required microbiological confirmation of *M tuberculosis*; probable tuberculosis required a chest radiograph suggestive of the disease (lymphadenopathy, miliary pattern, pleural effusion, bronchial compression, or parenchymal infiltrate) plus at least one additional feature of tuberculosis; possible tuberculosis required either a chest radiograph suggestive of tuberculosis in the absence of any additional feature of the disease, or a chest radiograph not suggestive of tuberculosis but a diagnosis of tuberculosis by the treating clinician and the presence of at least two additional features of the disease. During follow-up we also collected data on local and systemic events related to vaccination, admissions to hospital for all causes and for tuberculosis, and mortality.

### Sample size, interim analyses, and stopping rules

The trial was designed to evaluate whether the two routes of BCG administration were equivalent in preventing tuberculosis. The null hypothesis was that there would be a difference in the incidence of tuberculosis between the two groups of at least Δ, where Δ equalled 25%. A range of equivalence of within 25% was prespecified based on interpretation of a clinically meaningful difference associated with the different routes. We estimated the rate of tuberculosis at 2.5% per annum in this population. The birth rate in the study area was estimated to be 6302 per annum.^17^ The percentage of the birth cohort expected to receive BCG was estimated to exceed 85%, based on BCG coverage rates collected routinely by the regional health department.^18^ As the intervention was a single administration of the vaccine we anticipated the rate of withdrawal to be zero. The infant mortality rate was expected to be about 35 per 1000^19^ live births and the mortality rate in the second year to be 9 per 1000 live births per annum.^20^ After consultation with local experts we assumed an out migration rate of less than 10%.

We calculated that a cohort of 12 000 infants, randomised in a 1:1 ratio and followed up for two years, would yield enough cases in each arm to assess equivalence in the range 2.5% (SD 25%) per annum with a power (1−β) of 0.80 and at a level of significance (α) of 0.05.

We carried out two interim analyses, 14 and 29 months after the first enrolment when 2674 (22.9%) and 7685 (65.8%) of the total 11 680 infants, respectively, had been enrolled. On each of these occasions the results were presented to an independent international data safety monitoring committee of seven members, who considered whether the study should continue, on the basis of prespecified stopping rules. No rules were applied.

### Randomisation, sequence generation, allocation concealment, implementation, and masking

The study statistician generated the randomisation sequence in Microsoft Excel. Infants were allocated to route of BCG vaccine according to the week of birth. The sequence was balanced in eight week blocks (four intradermal, four percutaneous). Allocation concealment and masking did not take place. Participants were enrolled by midwives and study counsellors based at maternity units who were aware of the week’s allocation.

### Statistical analysis

The primary end point was the cumulative two year incidence of tuberculosis. The numerator for the calculation was all infants meeting the criteria for tuberculosis; the denominator was all randomised infants. Each infant was counted once only and in the case of multiple referrals for evaluation for tuberculosis we selected the most severe diagnosis as the outcome. We calculated exact binomial confidence intervals for the cumulative incidence rates. To account for two partially unblinded interim analyses, which used 0.0005 and 0.014% significance levels, respectively, we used a 4.5% significance level in the final analysis, following a previously published method.^21^ Equivalence was shown if the 95.5% confidence intervals for the incidence rates in the percutaneous group fell within 25% of those for the intradermal group. We further compared the cumulative incidence rates for the groups using risk ratios and corresponding Fisher’s exact confidence interval.

We compared the groups for baseline personal and clinical characteristics using two way analysis of variance, with vaccine group and study centre as factors for continuous variables, and the Cochran Mantel-Haenszel test, controlling for study centre, for categorical variables. We used cumulative incidence proportion and exact 95% binomial confidence interval to summarise the number of infants with at least one serious adverse event, the number of grade 3 and 4 vaccine related adverse events, and the number of deaths and compared these using risk ratios with corresponding 95% confidence intervals.

## Results

Overall, 13 338 pregnant women or those who had recently delivered were approached to enrol their infants in the trial (fig 2). In total, 11 680 infants were randomised and vaccinated. Reasons for exclusion included antenatal and perinatal complications—for example, stillbirth, prematurity, or intrauterine growth retardation and perinatal infection—as well as women who had agreed to the trial but declined after enrolment. Overall, 5905 infants received intradermal BCG and 5775 percutaneous BCG. During the three months after birth 4851 infants were followed up for adverse events after immunisation and all of these were seen at least once, some more than once. All 11 680 infants vaccinated were passively followed up for tuberculosis, morbidity, and mortality and were thus included in the primary intention to treat analyses.

**Figure fig2:**
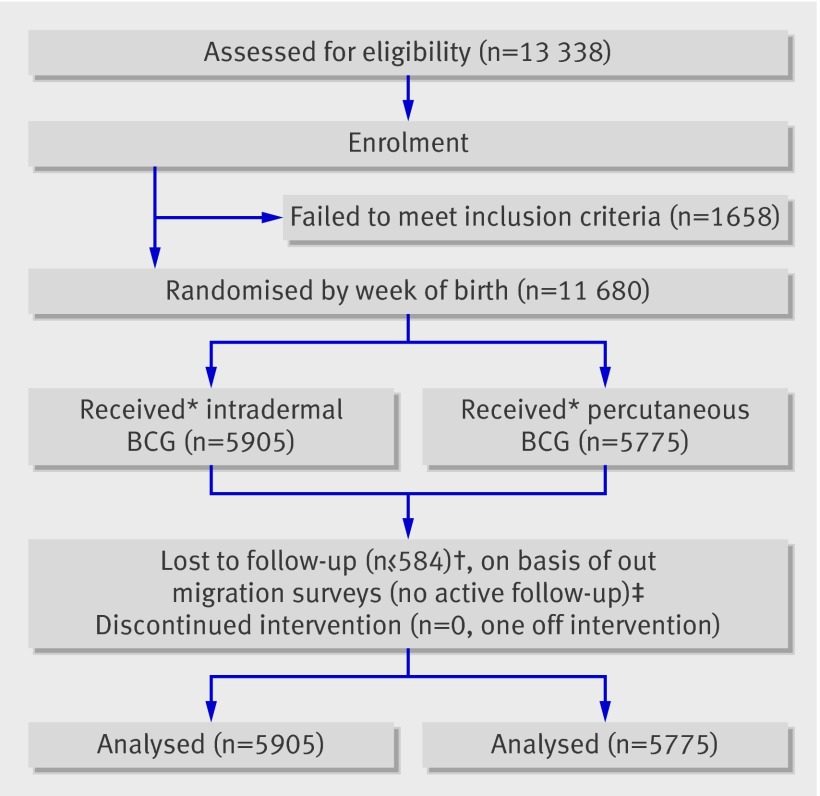
**Fig 2** Participant flow. *Some infants received vaccine through the alternative route to that allocated for operational reasons—for example, born shortly before midnight but vaccinated next morning, after allocation route changed. Sixty three (0.54%) infants received BCG by non-allocated route; 29 (0.50%) in intradermal group, 34 (0.58%) in percutaneous group. †10% random sample surveyed and less than 5% could not be traced. ‡Differential loss to follow-up by group cannot be calculated as out migration surveys did not collect this information

### Protocol violations

Two hundred and thirty violations of the protocol were recorded. Eight related to consent, 142 to the infant being vaccinated more than 24 hours after birth, 63 to infants assigned to one route but vaccinated by the alternative route, six to infants vaccinated after the trial had finished, and 25 to other aspects of the vaccination process. These infants were included in the intention to treat analysis. Protocol violations were reported to the data safety monitoring committee and relevant research ethics committees. When the violation had clinical implications, the infant was followed up appropriately and referred for further assessment if necessary.

### Recruitment and baseline data

The recruitment period spanned three years and four months, from 26 March 2001 to 31 July 2004. The follow-up period lasted until 31 July 2006 or until the infant reached 2 years of age, whichever occurred first. Table 1 shows the baseline personal and clinical characteristics of the infants by vaccination group.

**Table 1 tbl1:** Comparison of baseline personal and clinical characteristics of infants allocated to receive BCG vaccination by the intradermal route or percutaneous route. Values are numbers (percentages) unless stated otherwise

Factor	Intradermal BCG (n=5905)	Percutaneous BCG (n=5775)
Person years of exposure*	14 576.9	14 376.7
Boys	3026 (51.2)	2917 (50.5)
Mean (SD) gestational age at birth (weeks)	38.57 (2.23)	38.54 (2.24)
Preterm births (<37 weeks)	1038 (17.6)	981 (17.0)
Caesarean sections	645 (10.9)	635 (11.0)
Multiple births	48 (0.8)	70 (1.2)
Mean (SD) birth weight (g)	2930 (530)	2930 (530)
Low birth weight (≤2500 g)	1234 (20.9)	1185 (20.5)
Mean (SD) maternal age at infant’s birth (years)	25.70 (6.45)	25.65 (6.38)

*Time from vaccination date to date of diagnosis of most severe tuberculosis.

### Numbers analysed

All 11 680 vaccinated infants were analysed—5775 in the percutaneous group and 5905 in the intradermal group. These infants contributed person time to the denominators of the calculated rates. Loss to follow-up was assumed to be evenly split between both routes. It was not adjusted for in the calculation of incidence rates.

All infants were analysed in the group to which they has been randomised, regardless of vaccine received. Because enrolment into the trial was defined by the infant being vaccinated and because the intervention was one off and irreversible, it was not expected that a per protocol analysis would differ in any significant way from the primary, intention to treat analysis. No significant difference was found. For example, the difference between the intradermal and percutaneous groups in the two year cumulative incidence of definite, probable, and possible tuberculosis in the per protocol analysis was 0.39% (95.5% confidence interval −1.30% to 0.53%) compared with 0.36% (−1.27% to 0.54%) in the intention to treat analysis.

### Outcomes and estimation

The primary outcome of the trial was documented *M tuberculosis* infection or radiological and clinical evidence of tuberculous disease, expressed as the cumulative incidence of definite, probable, or possible tuberculosis over the two years of follow-up.

### Primary outcome

Equivalence was found between the routes using an end point of definite, probable, or possible tuberculosis (table 2 and fig 3).This is because the upper and lower bounds of the 95.5% confidence intervals around the point estimate of the rate in the comparator group (percutaneous) lay wholly within the interval bounded by the point estimate of the rate in the reference group (intradermal) plus or minus 25% (4.58% to 7.64%).^22^

**Table 2 tbl2:** Number of infants with tuberculosis by route of BCG administration, diagnosed within two years of birth in case verification ward

Classification	Intradermal BCG (n=5905)	% (95.5% CI)	Percutaneous BCG (n=5775)	% (95.5% CI)	Difference (intradermal−percutaneous)
Definite tuberculosis	89	1.51 (1.20 to 1.86)	83	1.44 (1.14 to 1.79)	0.09 (−0.37 to 0.54)
Probable tuberculosis	95	1.61 (1.30 to 1.97)	118	2.04 (1.69 to 2.51)	−0.42 (−0.93 to 0.08)
Possible tuberculosis	178	3.01 (2.58 to 3.49)	174	3.01 (2.58 to 3.50)	−0.03 (−0.68 to 0.61)
Definite, probable, and possible tuberculosis	362	6.13 (5.52 to 6.79)	375	6.49 (5.86 to 7.18)	−0.36 (−1.27 to 0.54)

**Figure fig3:**
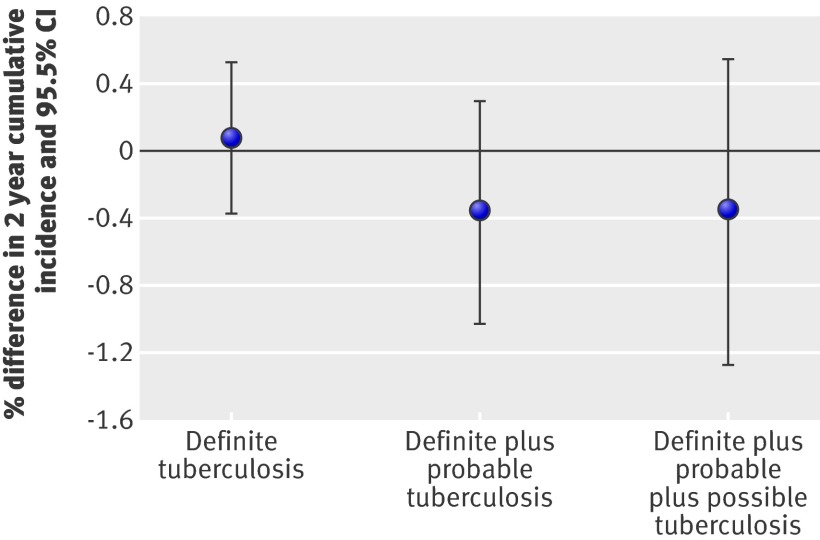
**Fig 3** Percentage difference (95.5% confidence intervals) in cumulative incidence of tuberculosis over two years by end point

Analysis of definite and probable cases only and definite cases alone just failed to show equivalence: in the first case the upper bound of the 95.5% confidence interval around the point estimate of the cumulative incidence in the percutaneous group lay marginally above the 25% band and in the second case marginally below the band.

Few cases of disseminated tuberculosis were detected and there were no statistically significant differences by route of vaccination. Three cases of tuberculous meningitis occurred in the intradermal group and one in the percutaneous group (risk difference 0.03%, 95% confidence interval −0.03% to 0.10%). No cases of miliary tuberculosis occurred in the intradermal group but one occurred in the percutaneous group (risk difference −0.02%, −0.05% to 0.02%). One case of abdominal tuberculosis and one case of spinal tuberculosis occurred in the percutaneous group. No statistical comparisons were made because of the small number of infants.

Only those infants who were suspected of having tuberculosis and who were admitted to the ward for investigations were tested for HIV. Thirty one of 1576 (1.97%) tested positive (enzyme linked immunosorbent assay, confirmed by polymerase chain reaction if the infant was less than 18 months old); 9.7% of these were diagnosed as having definite tuberculosis, 35.5% as having probable tuberculosis, and 16.1% as having possible tuberculosis.

### Secondary outcomes: adverse events

Overall, 2180 infants were admitted to hospital. The cumulative incidence was 18.7% (1091 (18.5%) in intradermal group, 1089 (18.9%) in percutaneous group): risk ratio 0.98 (95% confidence interval 0.91 to 1.06).

Twenty one grade 3 and one grade 4 vaccine related adverse reactions occurred. Of these, 16 were keloids (10 in intradermal group, six in percutaneous group, risk ratio 1.63, 0.59 to 4.48) and five were cases of suppurative axillary lymphadenitis (two in intradermal group, three in percutaneous group, risk ratio 0.65, 0.11 to 3.90), two of which progressed to breakdown with the formation of a draining sinus. None of the five infants was infected with HIV. The lesions resolved with conservative management without the use of systemic or local antituberculosis drugs.

One case of disseminated BCG disease was diagnosed in an infant who had received percutaneous BCG. The diagnosis was made after culture of a mycobacterium from gastric washings, which was subtyped as *M bovis* BCG substrain Tokyo 172. The infant was not infected with HIV, but had evidence of a primary immune deficiency, which was investigated at a specialist clinic at a tertiary academic hospital.

One hundred and eighty six infants died (102 (1.73%) in intradermal group, 84 (1.45%) in percutaneous group, risk ratio 1.19, 0.89 to 1.58). The cumulative incidence was 1.59%. None of these deaths was considered to be related to the intervention. Additionally, no deaths were considered to be caused by tuberculosis, although four were “associated with” the disease, two in the intradermal group and two in the percutaneous group. The causes of death are covered elsewhere.^23^

## Discussion

In a randomised trial we found equivalence in the cumulative incidence of definite, probable, and possible tuberculosis in infants before age 2 years who received either intradermal BCG or percutaneous BCG at birth.

The diagnosis of tuberculosis in infants is difficult. The most robust definition of tuberculosis in this age group is definite or probable. This was not used as the primary outcome in this trial for several reasons. Tuberculosis in young children in countries with a high burden of tuberculosis is generally not diagnosed on the basis of cultures owing to unavailability. Therefore the incidence of culture positive tuberculosis in a community trial such as this was difficult to predict. It was always intended that several case definitions and end points would be explored in the analysis. No recent and few older trials of tuberculosis vaccines in young children and infants were available on which to model this trial. It is encouraging that there is some consistency in the primary result regardless of the case definition used. Part of the reason for doing the trial was to develop a robust case definition and end point for use in future phase III trials of new tuberculosis vaccines.

For a variety of reasons the intradermal route for administering BCG vaccine is currently the most widely used worldwide and is the only method advocated by WHO.^5^ Although evidence is scant, a 2004 review of studies comparing the intradermal route with the percutaneous route found that percutaneous BCG is less efficient in stimulating interferon-γ production by Th1 lymphocytes and suggested that intradermal BCG should be recommended.^5^ This route is, however, technically more challenging and as such requires more training of operators and more intensive quality control of the immunisation programme. In many areas of the world, including several countries with a high burden of tuberculosis, these extra resources are not available, with the result that intradermal BCG may be given using incorrect techniques and after little or suboptimal training. Our results support allowing national or regional programmes for tuberculosis control to decide which route to use, basing their decision on other considerations such as logistics, training, economic factors, and product availability.

The rates of tuberculosis were high, given the documented quality controlled vaccination of the infants. Regardless of which route worked better, neither worked particularly well in preventing pulmonary disease, with over 6% of properly vaccinated infants developing definite, probable, or possible tuberculosis over the two years after birth. Even if possible cases were discounted, because of less confidence in the diagnosis, the rate is over 3%. A counter argument is that we have no way of knowing what the rate would be in the absence of BCG vaccination since placebo controlled trials are not possible, and thus we have no way of determining overall vaccine efficacy.

We found few cases of disseminated extrapulmonary tuberculosis. Disseminated disease may comprise over 25% of all cases of tuberculosis in children aged 0-14 years^24^ ^25^ and tuberculous meningitis in the Western Cape Province of South Africa has been reported to occur at rates of 0.03% per year in infants aged less than 1 year and 0.02% per year in children aged 1-4 years.^26^ We detected four cases of tuberculous meningitis, giving a cumulative incidence over two years of 0.03% or 0.015% per year, about half of that expected. Miliary tuberculosis is said to occur in around 2.7% of children with active tuberculosis.^27^ Given our calculated cumulative incidence of all tuberculosis over two years of 6.31%, we should have seen a cumulative incidence of miliary tuberculosis of around 0.17% over two years, or 20 cases. We, however, saw one case, giving a cumulative incidence over two years of 0.009%, about 20 times less than expected.

There are several possible explanations for this. Firstly, the process of carrying out the trial brought considerable resources into the study site that had not been available previously. Some of these resources were used to trace contacts of adults with tuberculosis, to intensively evaluate them for tuberculous disease, and to refer them for prophylaxis or treatment if they were infected or diseased. Because of this, many infants were detected soon after they had been infected with *M tuberculosis*, before they could develop clinical disease. All were referred for a course of prophylactic treatment (three months of daily rifampicin plus isoniazid), and this would have prevented most of them developing severe pulmonary or disseminated disease. Tracing and correctly managing contacts of children with tuberculosis will remain vital parts of all tuberculosis control programmes until and even after a more effective vaccine becomes available.

Secondly, our ability to detect cases of disseminated disease was perhaps suboptimal and we possibly missed cases and deaths due to such disease. Health providers in South Africa are, however, well aware of the condition and we think it is unlikely. Thirdly, our cases of pulmonary tuberculosis may not have been “real cases”—we were identifying infants with subclinical disease but positive culture results, some of whom would possibly, if left, have self cured. All cases, however, met internationally accepted definitions of paediatric tuberculosis and all would, if seen purely in a clinical setting in South Africa, have been placed onto full chemotherapy (all were referred for this reason). Fourthly, the “missing” cases of severe and disseminated disease may have been prevented by a more rigorous BCG immunisation programme and better quality control. Fifthly, our sample was biased towards more healthy newborn infants—those ineligible to receive routine BCG vaccination within 24 hours of birth were excluded from participation. This may at the same time have excluded those most likely to develop disseminated disease. Lastly, our estimates of the expected numbers of infants with miliary tuberculosis and tuberculous meningitis may be wrong. 

Overall, 186 infants died. A mortality surveillance system was developed to identify and document the causes of death as far as possible. Pneumonia, gastroenteritis, and septicaemia were among the commonest causes of mortality, and important underlying causes included HIV/AIDS, prematurity, low birth weight, and malnutrition. In this cohort, mortality was largely due to infectious diseases. For several reasons, however, many deaths could only be categorised as “sudden unexplained” or as “ill defined,” illustrating the difficulty of doing accurate mortality surveillance in field trials in developing countries. This has important implications for future phase III efficacy trials carried out for product registration.

More than one in six infants (n=2180) were admitted to hospital at least once during the course of the trial, emphasising the amount of comorbidity in this community. The number of adverse vaccine reactions was, however, relatively low with just five cases of axillary lymphadenopathy (0.4%) and one case of disseminated BCG infection (0.01%).

### Technical issues

We were not able to show equivalence in the cumulative incidence of definite and probable tuberculosis or of definite tuberculosis only between the groups, and in the definite only group the difference was in the opposite direction, with the rate in the percutaneous group being marginally lower. We believe that the explanation for this observation lies with the sample size of the study, which was powered to detect a difference using the most sensitive and least specific end point and that had our numbers been larger we would have shown equivalence for both the alternative, more specific end points. The apparent reversal of direction was, we feel, a function of chance. The finding does, however, underline the importance of having a clear and robust end point and being able to define what a “case” of tuberculosis is for the purposes of a phase III trial of any novel tuberculosis vaccine.

### Potential sources of bias and imprecision and study limitations

**Allocation concealment—**Mothers of potentially eligible infants were asked to decide whether to enrol them before being informed of the route of vaccination for the current week. Refusal rates were low and no greater for one route than the other. Eleven of 11 691 women gave consent before delivery but refused to confirm consent after delivery.

**Primary outcome assessment—**It was not possible to blind field staff assessing outcomes such as tuberculosis and adverse reactions to the route of vaccination. Chest radiography and microbiological tests such as smears and cultures were, however, always carried out and reviewed in a blinded fashion, and the final categorisation of cases was done blinded.

**Laboratory contamination—**A quality control programme was established during the trial, in which dummy sterile specimens were regularly submitted to the microbiology laboratory along with routine specimens. As the programme was not in place from the start and for other reasons, we cannot definitively exclude cross contamination as a source of some positive culture results. It is possible that we overestimated the absolute rates of culture positive tuberculosis in the cohort.

**Review of chest radiographs—**Reviewers were asked to classify *x* ray films using standard criteria and without access to the clinical details of the infant. When there was disagreement between reviewers we accepted a two thirds majority as definitive.

**Assessment of exposure to tuberculosis—**The caregivers’ recall of exposure to contacts as a means of establishing epidemiological linkage may be inaccurate. The significance of a tuberculin skin test result is difficult to gauge in a population where malnutrition and other causes of suppressed cellular immunity are rife, BCG vaccination is close to universal, and exposure to non-tuberculous mycobacteria common.

**Clinical diagnosis of tuberculosis—**The clinical diagnosis of paediatric tuberculosis is subjective and consequently imprecise given that the symptoms and signs of tuberculosis in children are non-specific.

**External validity of study findings—**We enrolled about two thirds of the region’s birth cohort. Those infants who were not enrolled might have come from families that were poorer and more likely to have tuberculosis. We included only infants who were eligible for routine BCG vaccination at birth. Our results may therefore be less generalisable, specifically to groups at higher risk of tuberculosis than our study cohort—those with an annual risk of infection of more than 4% and those that include large proportions of preterm, low birthweight infants.

**Strain of vaccine—**Many strains of BCG exist. We compared two routes for giving a single substrain of BCG (Tokyo 172). It is possible that some strains of BCG are more efficacious than others in preventing tuberculosis.^14^ ^15^ ^28^ ^29^ Our finding of an equivalent incidence of tuberculosis between routes may not be generalisable to other substrains of BCG.

**Children diagnosed outside the case verification ward—**One hundred and sixteen children were diagnosed as having tuberculosis outside the case verification ward: 63 of these were in the intradermal group and 53 in the percutaneous group. Children with tuberculosis who were not detected by community surveillance and who were investigated and diagnosed as having tuberculosis during a routine hospital admission were likely to have more advanced tuberculosis, including meningitis and miliary disease, but were likely to have more missing diagnostic data. Therefore, for the purpose of the primary analysis, we included only those children diagnosed as having tuberculosis on the ward. The ward admitted infants throughout the trial, but we included only children investigated for tuberculosis before their second birthday in the primary analysis. Retrospective sensitivity analyses were done that included all infants with a diagnosis of tuberculosis while on the ward and in the community and all infants investigated for tuberculosis before and after age 2 years. These analyses did not significantly change the relative efficacy estimates.

Despite all the potential limitations of the study, we believe that none should have significantly influenced the derived relative efficacy estimates.

The trial was not designed to provide an accurate estimation of the relative reactogenicity of the two routes. This was because surveillance was largely passive and infants who develop “minor” adverse events after immunisation do not generally present to healthcare facilities in South Africa. Only those infants who experienced major reactions, such as suppurative lymphadentitis, could be identified.

### Conclusion

In conclusion, the intradermal and percutaneous routes for BCG vaccination were equivalent for efficacy and safety. In addition to this some important issues will need to be overcome before phase III efficacy trials of new tuberculosis vaccines in young children can be carried out. This study allowed us to start putting together the necessary infrastructure for such trials, at least at one field site. Our results have important implications for tuberculosis control programmes and vaccination programmes that still advocate the use of intradermal BCG.

**What is already known on the topic**

Vaccination of newborn infants with BCG provides variable and incomplete protection against tuberculosis
WHO and most countries advocate the intradermal route for administering BCG vaccine
No study to date has provided clear evidence that one route of vaccination is safer, more immunogenic, or more efficacious than the other

**What this study adds**

The intradermal route for administering BCG vaccine to infants and young children was as efficacious as the percutaneous route for preventing tuberculosis
The two routes were equivalent in terms of safety

## Notes

Data Safety Monitoring Committee: Prakash Jeena (chair), Christine Grady, Lesley Henley, Linda Gail Bekker, Lisa Saiman, Mike Stoto, Neil Cameron, and Nicol Coetzee. Expert clinical panel: Maurice Kibel, John Burgess, and Peter Donald. Expert radiology panel: John Burgess, Maurice Kibel, and Robert Gie. The South African BCG trial team consisted of Andre Burger, Angela Malone, Anne Marie Demers, Ashley Veldsman, Aysel Gumusboga, Carolyn Kewley, Deon Minnies, Desire Michaels, Elmarie Simon, Gilla Kaplan, Heather Zar, Karen Iloni, Lea Denation, Lesley Workman, Linda van der Merwe, Marcel Behr, Marie Buchanan, Martien Borgdorff, Marijke Geldenhuys, Maurice Kibel, Michele Tameris, Monique Hanslo, Ronel Shepherd, Simon Schaaf, Siziwe Mawu, Sizulu Moyo, Stefanie Abraham, Suzanne Verver, Sylvia Mlanjeni, and Veronica Dirks. We thank Takeru Hashimoto, Akira Koyama, Naoki Nakada, and the management of the Tokyo BCG laboratory, Japan; Frans Krige and management of health department, Boland Overberg Region, Lizette Phillips and management of Brewelskloof Hospital, Worcester, and the participants.

Ethical approval: The study protocol was approved by the research ethics committee of the Faculty of Health Sciences, University of Cape Town and the research in human subjects committee of the Aeras Global TB Vaccine Foundation. The trial was not externally monitored but two audits were done during the enrolment period, on behalf of the sponsor, by an independent contract research organisation, Triclinium, Johannesburg, South Africa.

Provenance and peer review: Not commissioned; externally peer reviewed.

## Notes

**Cite this as: ***BMJ* 2008;337:a2052
